# PKC-Dependent Signaling Pathways within PAG and Thalamus Contribute to the Nitric Oxide-Induced Nociceptive Behavior

**DOI:** 10.1155/2013/471378

**Published:** 2013-08-21

**Authors:** Nicoletta Galeotti, Carla Ghelardini

**Affiliations:** Department of Neuroscience, Psychology, Drug Research and Child Health (NEUROFARBA), Section of Pharmacology and Toxicology, University of Florence, Viale G. Pieraccini 6, 50139 Florence, Italy

## Abstract

Nitric oxide (NO) is an important molecule involved in nociceptive processing in the central nervous system. The release of NO within the spinal cord has long been implicated in the mechanisms underlying exaggerated pain sensitivity, and administration of NO donors can induce hyperalgesia. To elucidate the supraspinal mechanism responsible for NO-induced nociceptive hypersensitivity, we investigated the modulation of protein kinase C (PKC) and downstream effectors following treatment with the NO donors nitroglycerin and sodium nitroprusside. Both compounds induced a prolonged cold allodynia and heat hyperalgesia, increased levels of c-Fos and IL-1*β*, and activated NF-*κ*B within periaqueductal grey matter and thalamus. Simultaneously, an increased expression and phosphorylation of PKC *γ* and *ε* were detected. To clarify the cellular mechanism involved in the NO-induced hypernociception, we examined the expression of transcription factors that act as PKC downstream effectors. A dramatic hyperphosphorylation of CREB and STAT1 was observed. The i.c.v. administration of the PKC blocker calphostin C prevented the NO-induced hypernociception, the hyperphosphorylation of CREB and STAT1, and partially reduced NF-*κ*B activation. Conversely, the increase of IL-1*β* was unmodified by calphostin C. These results suggest the relevance of cerebral PKC-mediated CREB and STAT1 activation in the NO donor-induced nociceptive behavior.

## 1. Introduction

Nitric oxide (NO) is a molecule importantly involved in pain processing within central nervous system. The release of NO within the spinal cord has long been implicated in the mechanisms underlying exaggerated pain sensitivity [1]. A reduced nociceptive behavior was observed in inflammatory and neuropathic pain after intrathecal administration of NO synthase (NOS) inhibitors [2, 3] and after genetic deletion of the NOS isoforms [4–6]. In addition, NO donors induced thermal [7] and mechanical [8] hyperalgesia.

Studies on the possible mechanism of action of NO and subsequent steps in the NO-induced cascade show that NO signals by various mechanisms including cGMP synthesis, nitrosylation of ion channels, ADP-ribosylation, and the interaction with molecular oxygen and superoxide radicals to produce reactive nitrogen species that can modify a number of macromolecules [9]. A major signaling mechanism of NO in spinal nociceptive processing initiates with activation of NO sensitive guanylyl cyclase and subsequent cGMP production [10, 11]. It has been speculated that activation of cGMP-dependent protein kinase I (cGKI), in turn, is the major effector of NO-dependent cGMP synthesis in the spinal cord [12, 13] and that the pronociceptive effects of the NO are mediated by activation of cGKI [14, 15]. However, the possible activation of targets different from cGKI has been postulated on the bases of data showing the lack of antagonism of the pronociceptive effect of an NO donor by the cGKI inhibitor [8]. Conversely to the spinal mechanisms, little is known on the supraspinal events modulated by NO.

Protein kinase C (PKC) is a family of enzymes involved in pain modulation. PKC integrates numerous receptor pathways into final effectors that increase excitatory signaling and decrease inhibitory signaling, thus inducing pain [16]. Recently, it has been reported that PKC inhibitors reduce NO synthesis from IFN-*γ*-treated microglia and that specific PKC isoforms (i.e., *δ*) are able to regulate NF-*κ*B activation and iNOS expression in mouse peritoneal macrophages [17, 18]. We, hence, aimed to investigate the involvement of PKC isoforms in the nociceptive behavior induced by NO donors' administration. To better elucidate the NO signaling cascade, we detected the expression of the main downstream PKC effectors in the early and delayed events produced by NO donors. In the brain, NO has been proposed to be involved in synaptic plasticity or to act as a neurotoxin when produced in excess [19], but its cerebral role in pain modulation is not completely understood. We, then, focused on supraspinal events induced by administration of the NO donors nitroglycerin (GTN) and sodium nitroprusside (SNP), and experiments were conducted in brain areas involved in pain modulation, such as thalamus and periaqueductal grey matter.

## 2. Materials and Methods

### 2.1. Animals

Male Swiss albino mice (20–22 g) from the Morini (San Polo d'Enza, Italy) breeding farm were used. Ten mice were housed per cage (26 × 41 cm). The cages were placed in the experimental room 24 h before the test for habituation. The animals were fed a standard laboratory diet and tap water *ad libitum* and kept at 23 ± 1°C with a 12 h light/dark cycle, light on at 7 a.m. All experiments were carried out in accordance with the European Communities Council Directive of 24 November 1986 (86/609/EEC). All efforts were made to minimize animal suffering and to reduce the number of animals used.

### 2.2. Behavioral Testing

Animals were habituated to the experimental room and were investigated by observers blinded for treatment of the animals.

#### 2.2.1. Cold Plate

For assessment of cold allodynia, mice were placed on a cold plate that is maintained at a temperature of 4 ± 0.1°C. Reaction times (s) were measured with a stopwatch before and 1, 2, 4, and 6 h after administration of the NO donors. The time between placements of a mouse on the plate and licking or lifting of a hind paw was measured with a digital timer. An arbitrary cut-off time of 60 s was adopted. 

#### 2.2.2. Hot Plate

Mice were placed inside a stainless steel container, which was set thermostatically at 50.0 ± 0.1°C in a precision water-bath (KW Mechanical Workshop, Siena, Italy). Reaction times (s) were measured with a stopwatch before and 1, 2, 4, and 6 h after administration of the NO donors. The endpoint used was the licking of the fore or hind paws. An arbitrary cut-off time of 60 s was adopted. 

#### 2.2.3. Motor Coordination

The motor coordination was assessed by using the rota rod test. The apparatus consisted of a base platform and a rotating rod with a diameter of 3 cm and a nonslippery surface. The rod was placed at a height of  15 cm from the base. The rod, 30 cm in length, was divided into 5 equal sections by 6 disks. Thus, up to 5 mice were tested simultaneously on the apparatus, with a rod-rotating speed of 16 r.p.m. The integrity of motor coordination was assessed on the basis of the number of falls from the rod in 30 s. Those mice scoring less than 3 and more than 6 falls in the pretest were rejected (20%). The number of falls was measured before (pretest) and 2, 4, and 6 h after the administration of the NO donors. 

#### 2.2.4. Locomotor Activity

The locomotor activity was evaluated by using the hole-board test. The apparatus consisted of a 40 cm square plane with 16 flush mounted cylindrical holes (3 cm diameter) distributed 4 by 4 in an equidistant, grid-like manner. Mice were placed on the center of the board one-by-one and allowed to move about freely for a period of 5 min each. Two photobeams, crossing the plane from midpoint to midpoint of opposite sides, thus dividing the plane into 4 equal quadrants, automatically signaled the movement of the animal (counts in 5 min) on the surface of the plane (locomotor activity). Miniature photoelectric cells, in each of the 16 holes, recorded (counts in 5 min) the exploration of the holes (exploratory activity) by the mice. Experiments were performed 4 h after administration of the NO donors. 

### 2.3. Western Blot Experiments

Experiments were conducted on PAG and thalamus of naïve, vehicle-, GTN-, or SNP-treated mice.

#### 2.3.1. Preparation of Whole Cell Lysates, Membranes, and Cytosol Fractions

Mice were perfused transcardially with 0.9% NaCl. Brain areas to conduct western blotting experiments were collected 1, 2, 4, and 6 h after the GTN (10 mg/kg i.p.) or SNP (1 mg/kg i.p.) treatment. Mouse brains were dissected to separate specific areas. PAG and thalamus were homogenized in an homogenization buffer containing 25 mM Tris-HCl pH = 7.5, 25 mM NaCl, 5 mM EGTA, 2.5 mM EDTA, 2 mM NaPP, 4 mM PNFF, 1 mM Na_3_VO_4_, 1 mM PMSF, 20 *μ*g/mL leupeptin, 50 *μ*g/mL aprotinin, and 0.1% SDS. The homogenate was centrifuged at 9,000 ×g for 15 min at 4°C; the low speed pellet was discarded. The supernatant (whole cell lysate) was centrifuged at 100,000 ×g for 60 min at 4°C. The resulting supernatant was the cytosol fraction, and the pellet was resuspended in the homogenizing buffer containing 0.2% (wt/vol) Triton X-100. The homogenate was kept at 4°C for 60 min with occasional stirring and then centrifuged at 100,000 ×g for 60 min at 4°C. The resultant supernatant was used as membrane fraction. Protein concentration was quantified using Bradford's method (protein assay kit, Bio-Rad Laboratories, Milan, Italy). 

#### 2.3.2. Western Blot Analysis

Membrane homogenates (10–50**** *μ*g) made from PAG and thalamus regions of GTN-, SNP-, vehicle-treated and naïve mice were separated on 10% SDS-PAGE and transferred onto nitrocellulose membranes (90 min at 120 V) using standard procedures. Membranes were blocked in PBST (PBS containing 0.1% Tween) containing 5% nonfat dry milk for 120** **min. Following washings, blots were incubated overnight at 4°C with specific antibodies against PKC*γ* phosphorylated on Thr514 (pPKC*γ*, 1 : 1000 dilution), c-Fos (1 : 1000) (Biosource, Camarillo, CA, USA); PKC*γ* (1 : 1000); PKC*ε* (1 : 800); PKC*ε* phosphorylated on Ser729 (pPKC*ε*, 1 : 750); iNOS (1 : 250); STAT1 phosphorylated on Tyr701 (pSTAT1, 1 : 500); *β*-actin (1 : 1000 dilution) (Santa Cruz Biothechnology Inc, CA, USA); CREB (1 : 500) or CREB phosphorylated on Ser133 (pCREB, 1 : 500) (cell Signalling Technology). After being washed with PBS containing 0.1% Tween, the nitrocellulose membrane was incubated with goat anti-rabbit horseradish peroxidase-conjugated secondary antisera (1 : 10,000) and left for 1 h at room temperature. Blots were then extensively washed according to the manufacturer's instruction and developed using enhanced chemiluminescence detection system (Pierce, Milan, Italy). Exposition and developing time used was standardized for all the blots. Optical density measurements were performed by dividing the intensity of the bands by the intensity of the house-keeping protein *β*-actin or GAPDH, used as loading control, at each time point. Measurements in control samples were assigned a relative value of 100%. 

### 2.4. Animal Treatment

The NO donors nitroglycerin (glyceryl trinitrate (GNT)) (Bioindustria L.I.M., Italy), dissolved in 10% ethylene glycol in saline (0.9% NaCl), and sodium nitroprusside (SNP) (Sigma, Italy), dissolved in saline, were administered intraperitoneally (i.p.).

To investigate the role of PKC in the intracellular events modulated by NO donors, the PKC blocker calphostin C (Calbiochem, Milan, Italy) was used. Time-course experiments performed in our laboratory showed that the calphostin C effect peaked 1-2 h after intracerebroventricular (i.c.v.) administration. Animals were, then, divided into different treatment groups.

#### 2.4.1. Behavioral Testing

Vehicle, GTN, or SNP were i.p. administered, and pain threshold was evaluated 1 and 2 h after treatment. Following NO donors' administration, animals received a single i.c.v. injection of vehicle (0.5% DMSO) or calphostin C (0.2 *μ*g per mouse) 3 h after GNT or SNP treatment. The pain threshold was, then, evaluated at 4 h, in coincidence with the maximal hypernociceptive activity of NO donors, and at 6 h when the nociceptive behavior disappeared.

#### 2.4.2. Western Blot

Brain areas samples to perform experiments were removed 1, 2, 4, and 6 h after NO donors' administration. Animals were divided in two groups of treatment: (1) vehicle (0.5% DMSO) or calphostin C administered 10 min before NO donors injection and protein expression detected 2 h after GNT/SNP administration; (2) vehicle (0.5% DMSO) or calphostin C administered 3 h after NO donors and protein expression detected 4 h after GNT/SNP. 

Aspirin 40 mg/kg (Bayer AG, Leverkusen, Germany) was dissolved in 0.5% DMSO and administered i.p. 3 h after NO donor injection. Brain samples were collected at 4 h. 

Lipopolysaccharide (LPS) (60 mg/kg ip, Sigma, Italy) was used as positive control of IL-1*β* expression. Brain areas to conduct experiments were removed 6 h after LPS administration.

Intracerebroventricular (i.c.v.) administration was performed as previously described [20].

### 2.5. Statistical Analysis

Behavioral experiments results were given as mean ± SD. 10 mice per group were used. Two-way analysis of variance (ANOVA) followed by Bonferroni *post hoc* test was used for statistical analysis. Western blotting experimental results were given as the mean ± SEM of results obtained from 6–8 independent experiments. Analysis of variance (ANOVA) followed by Tukey *post hoc* test was used for statistical analysis.

## 3. Results

### 3.1. Nociceptive Hypersensitivity without Induction of Side Effects by NO Donors

**Figure 1:**
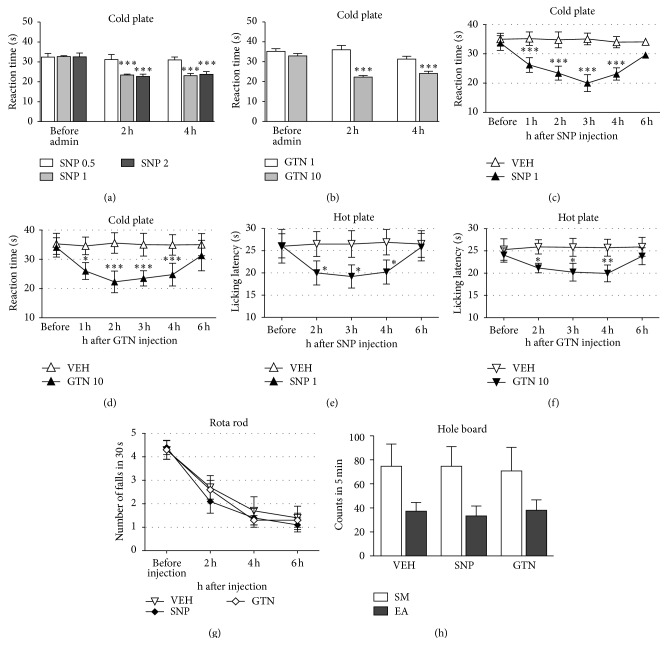
NO donors decrease the pain threshold without behavioral side effects. Administration of sodium nitroprusside (SNP, 0.5–2 mg/kg i.p.) (a) or nitroglycerin (GTN, 1–10 mg/kg i.p.) (b) induced cold allodynia, evaluated in the cold plate test. ^***^ *P* < 0.001 compared with before administration values. Time course studies showed a prolonged cold allodynia (c, d) and heat hyperalgesia, evaluated in the hot plate test (e, f) that disappeared 6 h after injection. GTN (10 mg/kg) and SNP (1 mg/kg) did not alter motor coordination (rota rod test) (g), spontaneous mobility, and inspection activity (hole board test) (h). ^*^ *P* < 0.05,  ^**^ *P* < 0.01,  ^***^ *P* < 0.001 compared with vehicle-treated mice (VEH).

The administration of SNP (0.5–2 mg/kg i.p.; Figure 1(a)) and GTN (1–10 mg/kg i.p.; Figure 1(b)) induced cold allodynia as revealed by the cold plate test. Time course studies showed a prolonged nociceptive behavior. The reaction times to the cold stimulus were reduced 1, 2, and 4 h after NO donors' administration. The pain threshold returned to control values 6 h after SNP (Figure 1(c)) or GNT (Figure 1(d)) injection. Following NO donor treatment, a thermal hyperalgesia was also observed in the hot plate test with a time course similar to the cold allodynia. Mice showed reduced licking latency values 2 and 4 h after administration whereas at 6 h the hyperalgesic effect disappeared (Figures 1(e) and 1(f)).

The reduction of the pain threshold was not accompanied by the induction of side effects. NO donors did not alter locomotor activity of treated animals at any time point, as indicated by the rota rod test results (Figure 1(g)). The spontaneous mobility (SM) and exploratory activity (EA) of mice treated with SNP and GTN were unmodified in comparison with the control group (hole board test; Figure 1(h)). 

### 3.2. Blockade of PKC Phosphorylation Prevented NO Donor-Induced Nociceptive Behavior

**Figure 2:**
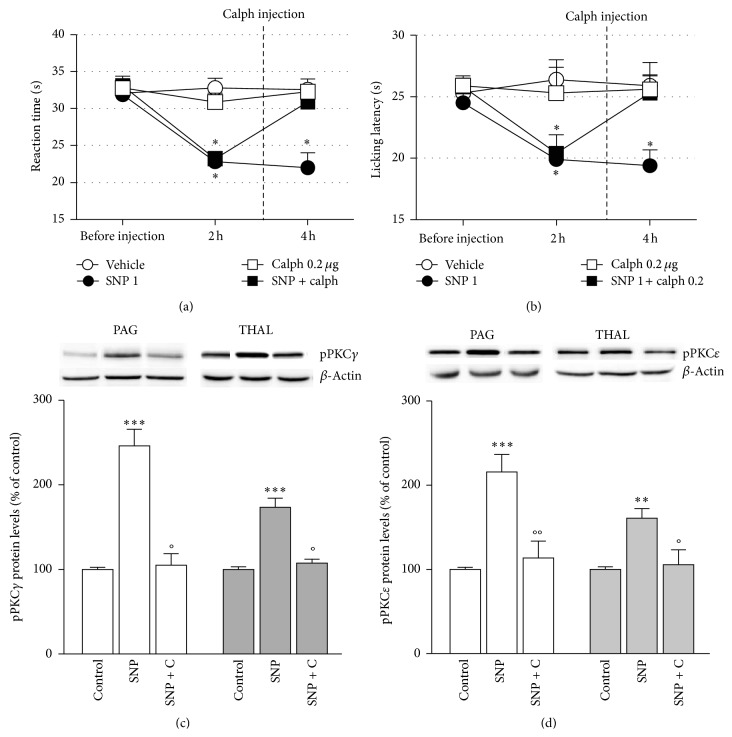
Allodynia and hyperalgesia produced by NO donors' administration underly a PKC-mediated pathway. The PKC blocker calphostin C (calph; 0.2 *μ*g per mouse i.c.v.) reversed cold allodynia (a) and heat hyperalgesia (b) induced by SNP. Calphostin C (c) completely prevented the SNP-induced upregulation of pPKC*γ* (c) and pPKC*ε* (d) within PAG and thalamus. ^*^ *P* < 0.05,^**^ *P* < 0.01,^***^ *P* < 0.001 compared with vehicle-treated control group; °*P* < 0.05,  °°*P* < 0.001 compared with SNP-treated group.

**Figure 3:**
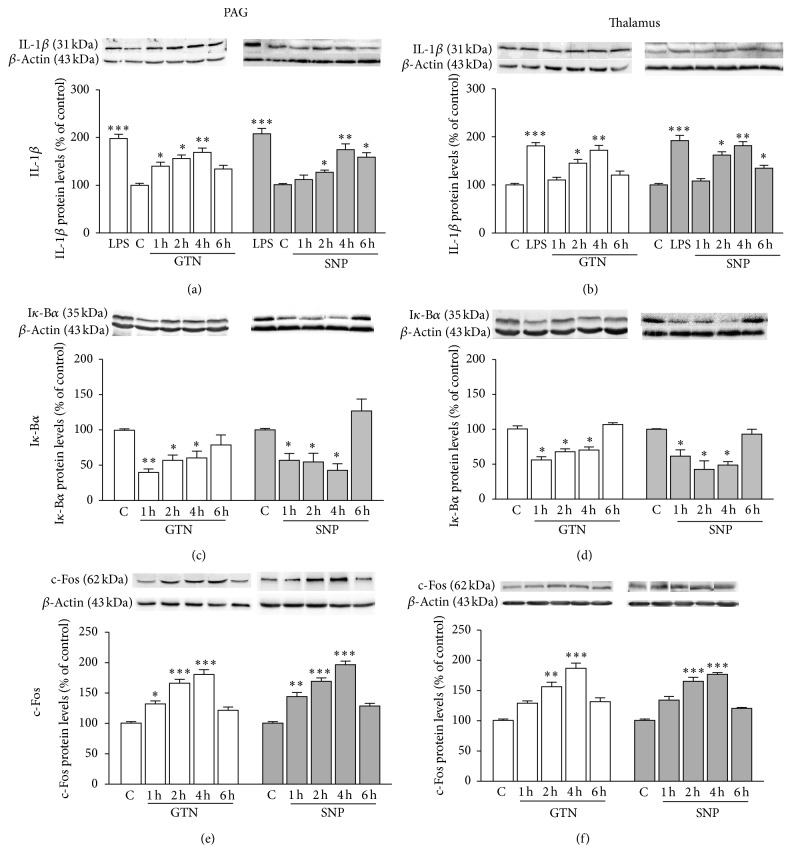
Cerebral modulation of IL-1*β* and NF-*κ*B by NO donors. GNT and SNP increased the expression of IL-1*β* within PAG (a) and thalamus (b) with a peak at 4 h after administration. LPS was used as positive control. NO donors produced a cerebral activation of the NF-*κ*B pathway as indicated by the reduction of I*κ*-B*α* levels in the PAG (c) and thalamus (d). NO donors increased c-Fos protein levels in the PAG (c) and thalamus (d) with a similar time course to the nociceptive hypersensitivity. ^*^ *P* < 0.05,^**^ *P* < 0.01,^***^ *P* < 0.001 compared with vehicle-treated control group (c).

The i.c.v. injection of the PKC blocker calphostin C (calph; 0.2 *μ*g per mouse) completely reversed the hypersensitivity to cold (Figure 2(a)) and heat (Figure 3(b)) stimuli induced by SNP leading to reaction times comparable to control values. Similar results were produced by GTN. Calphostin C, when administered alone, did not alter the mouse pain threshold suggesting the lack of any hyperalgesic/analgesic activity (Figures 2(a) and 2(b)). 

NO donors increased the levels of phosphorylated PKC*γ* (Figure 2(c)) and PKC*ε* (Figure 2(d)) within PAG and thalamus membrane fraction with a peak 2–4 h after treatment. Total PKC*γ* and PKC*ε* protein expressions were also increased 2 and 4 h after treatment with a similar time course for GTN and SNP. 

Calphostin C prevented the upregulation of pPKC*γ* and pPKC*ε*, indicating that the doses and administration schedule of calphostin C used in behavioral tests were ideal to block PKC activity.

Vehicles did not modify protein expression in comparison with naïve animals' protein content.

### 3.3. NO Donors Induced IL-1*β* Upregulation and NF-*κ*B Activation

To evaluate the supraspinal mediators involved in the induction of the NO-induced nociceptive behavior, we examined the expression of cellular components involved in inflammatory processes. In particular, IL-1*β* and NF-*κ*B were detected in homogenates of PAG and thalamus after administration of NO donors by immunoblotting technique. GTN and SNP induced a progressive increase of IL-1*β* up to 4 h after administration whereas at 6 h the IL-1*β* levels decreased. The maximal increase of IL-1*β* expression was comparable to that produced by LPS, used as positive control. A similar expression profile was detected in the PAG (Figure 3(a)) and thalamus (Figure 3(b)). 

The activation of the NF-*κ*B pathway was demonstrated by the reduction of the I*κ*-B*α* expression, the protein that constitutively inhibits NF-*κ*B. The decrease of I*κ*-B*α* peaked 1 h after administration with a prolonged effect, being significant up to 4 h after NO donors' administration (Figures 3(c) and 3(d)). 

c-Fos has been widely used as a marker of neuronal activation and pain. To further support the hypothesis of a supraspinal mechanism for the induction of a nociceptive behavior by NO, we detected the c-Fos protein content within PAG and thalamus, brain areas related to pain perception. A rapid and progressive increase of c-Fos expression within the PAG was observed after GTN and SNP administration (Figure 3(e)). Similarly, a robust increase of the c-Fos expression was detected 2 and 4 h after NO donor treatments within the thalamus (Figure 3(f)).

No difference between GTN and SNP was observed. Vehicles used did not modify the protein expression when compared with naïve animals' protein content.

### 3.4. Activation of CREB following NO Donors

**Figure 4:**
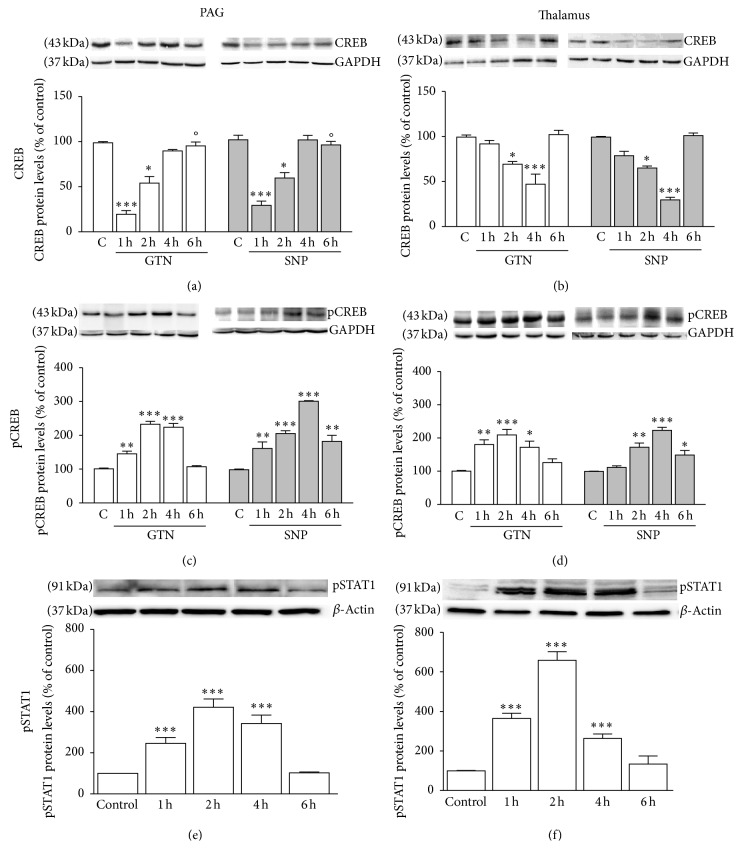
NO donors modulated CREB and STAT1 expression and phosphorylation. GNT and SNP modulated cerebral CREB protein levels as indicated by the CREB downregulation within PAG lysate (a) and thalamus (b). An upregulation of phosphorylated CREB (pCREB) following NO donors' administration was observed in the PAG (c) and thalamus (d). NO donors induced upregulation of phosphorylated STAT1 (pSTAT1). pSTAT1 protein levels were increased 1, 2 and 4 h after SNP treatment within PAG (e) and thalamus (f). ^*^ *P* < 0.05,^**^ *P* < 0.01,^***^ *P* < 0.001 compared with vehicle-treated control group (c).

GNT and SNP produced a drastic decrease of CREB levels in the whole cell lysates from PAG (Figure 4(a)). The reduction of CREB expression was evident 1 and 2 h after NO donors' administration; then the levels increased and returned to control values. A reduction of CREB protein content was also observed in the thalamus but with a slightly different time course: the decrease of the protein content was significant 2 h after administration, peaked at 4 h, and returned to control values at 6 h (Figure 4(b)). No difference between the effects produced by GTN and SNP was observed.

A robust increase of the phosphorylated form of CREB (pCREB) was detected in the total cell lysate from PAG (Figure 4(c)). This effect was significant 1 h after administration and peaked between 2 and 4 h, and then it drastically decreased. Similar results were obtained from experiment conducted on the total cell lysates from the thalamus (Figure 4(d)). 

Vehicles used did not modify protein expression when compared with naïve animals' protein content.

### 3.5. GTN and SNP Induced STAT1 Overexpression

A highly significant increase in the phosphorylated form of STAT1 (pSTAT1) was detected in the PAG (Figure 4(e)) and thalamus (Figure 4(f)) of SNP- and GTN- (data not shown) treated animals. The pSTAT1 contents increased 1 h after SNP administration and peaked at 4 h, and 6 h after treatment pSTAT1 levels returned comparable to control. In the thalamus the increase of the pSTAT1 expression showed a similar time course to the PAG, but the increase of pSTAT1 was greater than in the PAG. 

No difference between the effects produced by GTN and SNP was observed. Vehicles used did not modify protein expression when compared with naïve animals' protein content.

### 3.6. NO Donors Induce Allodynia and Hyperalgesia through a PKC-Dependent Mechanism

**Figure 5:**
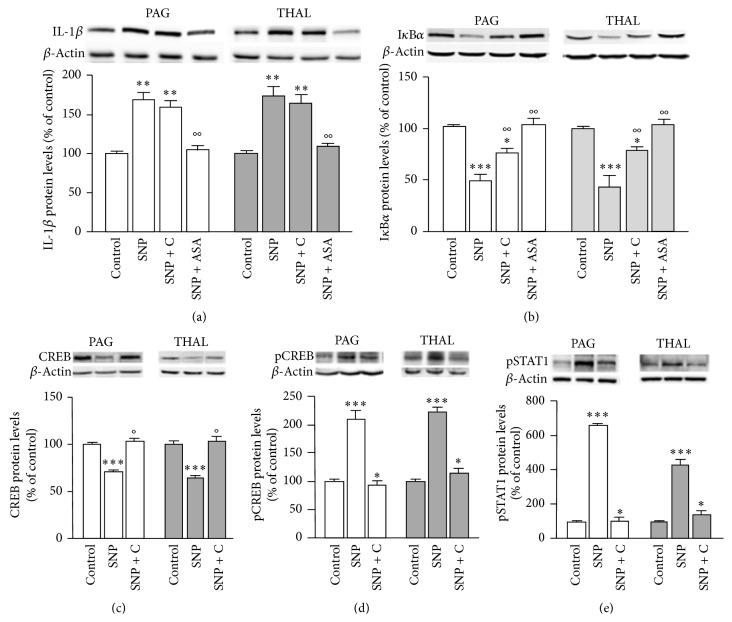
Activation of a PKC-independent and a PKC-mediated pathway by NO donors. (a) SNP-induced increase of IL-1*β* within PAG and thalamus (THAL) was unmodified by pretreatment with the calphostin C and prevented by aspirin (ASA). (b) The reduction of I*κ*-B*α* was partially prevented by calphostin C and completely restored by ASA. Calphostin C antagonized the downregulation of CREB (c) and prevented the increased expression of pCREB (d) and pSTAT1 (e). ^*^ *P* < 0.05,^**^ *P* < 0.01,^***^ *P* < 0.001 compared with vehicle-treated control group; °*P* < 0.05,°°*P* < 0.01 compared with SNP-treated group.

To investigate whether the cellular components modulated by NO donors represented downstream effectors of PKC, we detected their expression following calphostin C administration. The expression of IL-1*β* was unmodified by the PKC blocker whereas it was reduced by aspirin (ASA) in both cerebral areas (Figure 5(a)). The reduced levels of I*κ*-B*α* were only partially restored by calphostin C whereas ASA completely prevented the NF-*κ*B activation (Figure 5(b)). Conversely, the modulation of CREB and STAT1 expression was PKC-mediated. Treatment with calphostin C completely reversed the SNP-induced decrease of CREB protein levels (Figure 5(c)) and increase of pCREB (Figure 5(d)) and pSTAT1 within PAG and thalamus (Figure 5(e)). Calphostin C prevented the modulation of the expression of CREB, pCREB, and STAT1 1, 2, and 4 h after NO donors' administration. No effect was detected at 6 h where the levels of the above-mentioned proteins returned to control values.

## 4. Discussion

The systemic administration of the NO donors GTN and SNP in mice produced cold allodynia and heat hyperalgesia detectable 1 h after administration that peaked at 4 h. These results confirm and extend previous studies that described the induction of thermal hyperalgesia in rats after systemic administration of GTN with a similar time course [7]. 

To elucidate the mechanism responsible for the production of hyperalgesia, we investigated the role of the protein kinase C (PKC), a family of enzymes highly involved in pain modulation [16]. I.c.v. administration of the PKC blocker calphostin C prevented the NO donor-induced allodynia and hyperalgesia producing pain threshold values similar to those of the naïve animals. 

To evaluate the site of the pronociceptive action of NO donors, we detected the PKC expression within PAG and thalamus, cerebral areas highly involved in the modulation of pain perception. In GTN- and SNP- treated mice we detected a specific upregulation and increased phosphorylation of PKC*ε* and PKC*γ*, isoforms with a prominent role in the modulation of pain perception [16, 21, 22], concomitantly with the presence of allodynia and pain. It is known that PKC represents a second messenger pathway coupled to the induction of c-Fos, the protein product of the immediate early gene c-fos, widely used as marker of neuronal activation. The evaluation of the time-course of the c-Fos expression revealed a correspondence between c-Fos levels and the nociceptive behavior. Since c-Fos is also used as marker of pain [23], these results further confirm the presence of a neuronal activation related to the pain hypersensitivity detected by in vivo studies. These results have highlighted the importance of PKC in the nociceptive behavior induced by NO donors.

When can, therefore, hypothesize the presence of a PKC-mediated intracellular pathway activated by NO and responsible for the nociceptive behavior. To elucidate this pathway, we investigated PKC downstream effectors that might be involved in the induction of pain hypersensitivity, such as CREB and STAT1. CREB is a ubiquitously and constitutively expressed transcription factor. Its ability to activate transcription of the regulated gene critically depends on phosphorylation of a serine residue, Ser133, in its transactivation domain [24]. We detected a relevant influence on CREB phosphorylation by GTN and SNP. A robust increase in the phosphorylation of CREB has been observed in the PAG and thalamus with a peak at 2–4 h after GTN and SNP treatment, in coincidence with the presence of allodynia and hyperalgesia. The pCREB expression appeared to be PKC dependent since the PKC blocker calphostin C prevented the NO donors' modulation of this transcription factor. NO-induced CREB phosphorylation might contribute to the genesis of the hypersensitivity to noxious stimuli observed following NO donors' administration. This hypothesis is supported by observations that highlight a role for CREB in the modulation of pain sensation. In the early stages of inflammation [25] and sciatic nerve injury [26], the phosphorylation of CREB is increased in the dorsal horn through a mechanism involving several kinases, including PKC [27].

A dramatic increase in the expression of the phosphorylated form of STAT1 (pSTAT1) was observed following NO donors' injection. Within PAG and thalamus the pSTAT1 upregulation was detected beginning from 1 h up to 4 h after NO donors' administration. We can hypothesize an important role for STAT1 as cellular effector for NO donors. PKC appears to be an upstream modulator of  STAT1 since the PKC blocker calphostin C completely prevented the pSTAT1 increase. This hypothesis is supported by the observation that inhibition of classical PKC isoenzymes downregulate STAT1 activation in LPS-treated murine macrophages [28].

Many animal studies have shown that NO contributes to the sensitization during inflammatory pain. We, therefore, detected the supraspinal expression of important mediators of inflammation to determine their role in the NO-induced nociceptive behavior. GTN and SPN produced a progressive increase of IL-1*β* protein levels and activation of NF-*κ*B, as demonstrated by the reduction of I*κ*-B*α* protein levels. Conversely to CREB and STAT1, these mediators appeared not to be main PKC downstream effectors since pretreatment with the PKC blocker calphostin C did not modify IL-1*β* levels and only partially reduced I*κ*-B*α* protein levels. Since calphostin C completely prevented the NO donor-induced nociceptive behavior, we can exclude a major role for IL-1*β* and NF-*κ*B in the cerebral mechanisms responsible for the induction pain hypersensitivity.

Although GTN can accumulate and reach toxic levels in adipose tissue and lipid-rich organs such as brain, we can exclude that the hypersensitivity observed was related to an altered viability of mice. NO donors did not modify spontaneous mobility, inspection activity, and locomotor activity and were not endowed with visible behavioral side effects at any time point. As regards cardiovascular effect, both GTN and SNP systemically administered at the doses used in the present study induced moderate hypotension that lasted 40 and 80 min, respectively [29]. No altered cardiovascular parameter was observed 2 and 4 h after treatment [29], and we can suppose that pain hypersensitivity was not subsequent to a hypotensive effect.

These findings highlight the upregulation and increased phosphorylation of PKC*γ* and PKC*ε* as cerebral intracellular mechanism involved in the induction of pain hypersensitivity following NO donors' administration. The presence of a PKC-mediated pathway involving STAT1 and CREB as PKC downstream effectors has also been demonstrated. These cellular events were detected concomitantly with the presence of allodynia and hyperalgesia suggesting that these transcription factors might act synergistically to modulate pain perception. 

## Abbreviations

CREB: Cyclic AMP response element binding protein
GTN: Nitroglycerin
i.c.v.: Intracerebroventricularly
i.p.: Intraperitoneally
NO: Nitric oxide
NOS: NO synthase
PAG: Periaqueductal grey matter
PKC: Protein kinase C
SNP: Sodium nitroprusside
STAT: Signal Transducer and Activator of Transcription.
